# Efficient inhibition of amyloid fibrillation and cytotoxicity of α-synuclein and human insulin using biosynthesized silver nanoparticles decorated by green tea polyphenols

**DOI:** 10.1038/s41598-024-54464-4

**Published:** 2024-02-16

**Authors:** Behnaz Mirzaei-Behbahani, Ali Akbar Meratan, Beitollah Moosakhani, Mahya Mohammad-Zaheri, Zahra Mousavi-Jarrahi, Nasser Nikfarjam, Mohammad Bagher Shahsavani, Ali Akbar Saboury

**Affiliations:** 1 Department of Biological Sciences, Institute for Advanced Studies in Basic Sciences (IASBS), Zanjan, 45137-66731 Iran; 2 Institute of Biochemistry and Biophysics, University of Tehran, Tehran, 1417614335 Iran; 3 Department of Chemistry, Institute for Advanced Studies in Basic Sciences (IASBS), Zanjan, 45137-66731 Iran; 4 Protein Chemistry Laboratory (PCL), Department of Biology, College of Sciences, Shiraz University, Shiraz, 7196484334 Iran

**Keywords:** GTPs, GTPs-capped AgNPs, Amyloid fibril, Cytotoxicity, Human insulin, α-Synuclein, Biochemistry, Molecular biology, Neuroscience

## Abstract

Green tea polyphenols (GTPs), particularly epigallocatechin-3-gallate, stand out among natural small molecules screened for their ability to target protein aggregates due to their potent anti-amyloidogenic and neuroprotective activities against various disease-related peptides and proteins. However, the clinical applications of GTPs in amyloid-related diseases have been greatly limited by drawbacks such as poor chemical stability and low bioavailability. To address these limitations, this study utilized an Iranian green tea polyphenolic extract as a reducing agent to neutralize silver ions and facilitate the formation of silver nanoparticle capped by GTPs (GTPs-capped AgNPs). The results obtained from this study demonstrate that GTPs-capped AgNPs are more effective than free GTPs at inhibiting amyloid fibrillation and reducing cytotoxicity induced by amyloid fibrils of human insulin and α-synuclein (α-syn). This improved efficacy is attributed to the increased surface/volume ratio of GTPs-capped AgNPs, which can enhance their binding affinity to amyloidogenic species and boosts their antioxidant activity. The mechanism by which GTPs-capped AgNPs inhibit amyloid fibrillation appears to vary depending on the target protein. For structured protein human insulin, GTPs-capped AgNPs hinder fibrillation by constraining the protein in its native-like state. In contrast, GTPs-capped AgNPs modulate fibrillation of intrinsically disordered proteins like α-syn by redirecting the aggregation pathway towards the formation of non-toxic off-pathway oligomers or amorphous aggregates. These findings highlight polyphenol-functionalized nanoparticles as a promising strategy for targeting protein aggregates associated with neurodegenerative diseases.

## Introduction

Protein misfolding associated diseases encompass a large group of debilitating and uncurable pathogenic conditions in which a peptide or protein loss its native functional state^1^. The common hallmark of these diseases is the presence of abnormal fibrillar structures known as amyloid fibrils. Despite significant diversity in the amino acid sequence of peptides/proteins associated with amyloid fibril-related human diseases, these structures share common features including high content of β-sheets, solvent-exposed hydrophobic regions, and birefringence upon staining with Congo red (CR) and show a similar mechanism of self-assembly and aggregation^2^. Therefore, prevention of protein misfolding and amyloid fibril formation is considered as one the most effective approaches for treatment of amyloid-related diseases. For achieving this goal, several approaches have been suggested including (i) structural stabilization of the native form of amyloidogenic peptides and proteins, (ii) increasing the clearance rate of misfolded species, and (iii) interfering with the assembly process of proteins and/or redirecting the amyloid fibril formation toward non-toxic pathways^3,4^. This has led to extensive attempts to identify compounds with anti-amyloidogenic properties. Among them, naturally-occurring polyphenols, present extensively in foods and plants, are unique, not only by interfering in the various steps of the amyloid fibrillation process^5^, but due to their antioxidant properties leading to neuroprotective effects against amyloid cytotoxicity^6^. In fact, thanks to the presence of various functional groups, these molecules can target various biological molecules, and particularly proteins, thereby modifying their structure through modulating protein–protein interactions^7^. Among the reported natural polyphenols with the ability to inhibit amyloid fibril formation, green tea polyphenols (GTPs) have attracted prominent attention. Besides their antioxidant^8^, antimicrobial^9^, and antitumor activities^10^, extensive studies indicate that GTPs interact with different model and amyloidogenic peptides/proteins and efficiently inhibit or remodel their assembly process and reduce amyloid cytotoxicity^11–18^. However, disadvantages such as poor structural stability, rapid metabolism, low cellular uptake and thus inability to cross blood brain barrier (BBB)^19^, and decreased anti-amyloidogenic performance in phospholipid interfaces^20^, may significantly restrict the therapeutic applications of GTPs. Among strategies developed to overcome these drawbacks, a promising approach is using nanoscale materials, which have shown great efficiency in modulating amyloid fibrillation process and crossing BBB, where they target amyloid plaques^21–23^. It has been reported that the performance of different types of small molecules to inhibit amyloid fibril formation can be enhanced up to 100,000 times in their nanoparticle, compared to molecular, forms^24^. Recently, we also reported on the improved efficiency of polyphenols to modulate the fibrillation process as well as cytotoxicity of various proteins in their nanoforms using in vitro and in vivo systems^25–28^. These improved anti-amyloidogenic properties of nanoparticles may be attributed to their enhanced structural stability and more effective binding to protein species.

**Figure 1:**
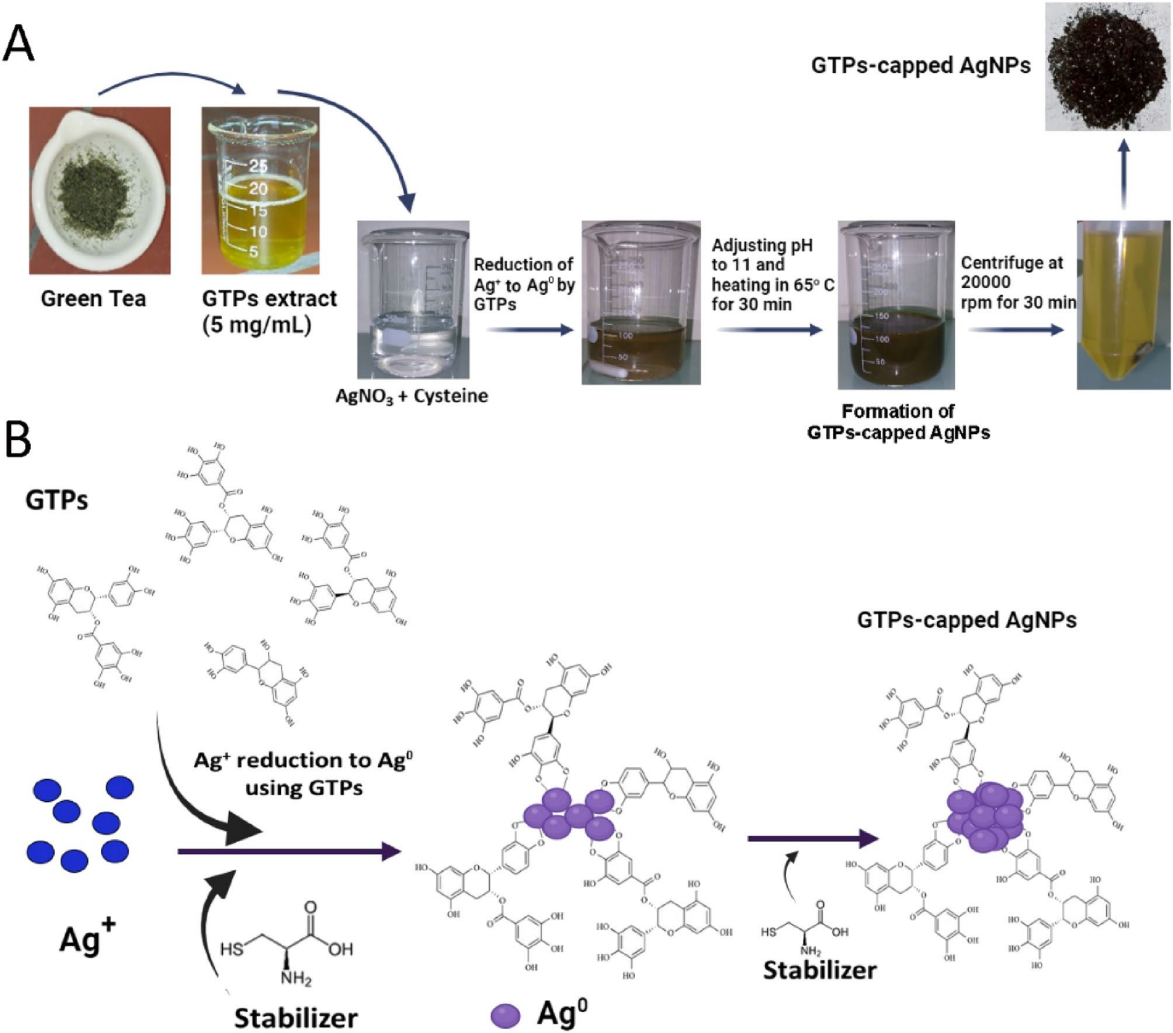
Schematic illustration showing (**A**) synthesis of GTPs-capped AgNPs and (**B**) a possible mechanism for reduction and stabilization of AgNPs mediated by GTPs.

Herein, this work presents a facile and low-cost method to biosynthesize silver nanoparticles stabilized by cysteine and capped with GTPs (GTPs-capped AgNPs) (Fig. 1). The GTPs extraction and GTPs-capped AgNPs synthesis were successfully confirmed by various spectroscopic and microscopic analyses. Then, the potency of GTPs and GTPs-capped AgNPs in modulating assembly process as well as cytotoxicity of amyloids arising from human insulin and α-synuclein (α-syn) was investigated by a range of amyloid fibril-specific techniques. Our findings clearly demonstrate that GTPs-capped AgNPs exhibit enhanced performance in inhibiting amyloid fibril formation, disintegrating preformed mature fibrils, and attenuating the cytotoxicity associated with amyloid fibrils. The improved anti-amyloidogenic properties of GTPs-capped AgNPs, as compared to their respective molecular form, can be attributed to their increased surface-to-volume ratio, which facilitates stronger binding to proteins and enhances their antioxidant activity.

## Results and discussion

### Extraction of GTPs, synthesis and characterization of GTPs-capped AgNPs

**Figure 2:**
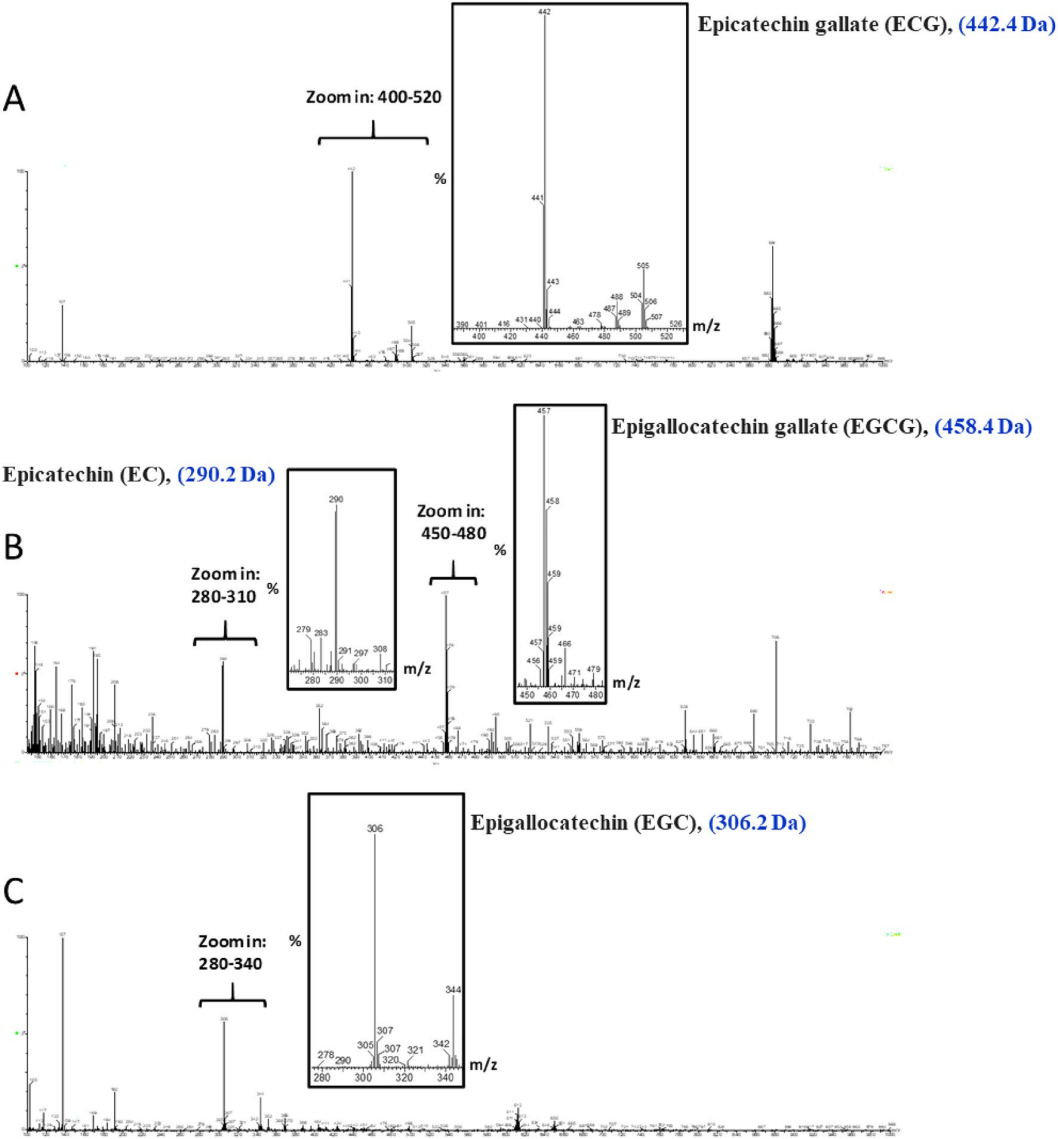
LC–MS profile of GTPs indicating the presence of four main catechins.

First, the successful extraction of GTPs was determined using liquid chromatography-mass spectrometry (LC–MS), indicating the presence of four catechins including epicatechin gallate (442.4 Da) (Fig. 2A), epicatechin (290.2 Da) and epigallocatechin gallate (458.4 Da) (Fig. 2B), and epigallocatechin (306.2 Da) (Fig. 2C) as the main tea polyphenols^29^. The strategy used for synthesizing GTPs-capped AgNPs is shown in Fig. 1. The mechanism for the synthesis of GTPs-capped AgNPs involves the reduction of Ag^+^ to Ag^0^ by GTPs and production of silver nanoparticles (AgNPs) by clustering Ag^0^s^30,31^. Solution color changing from colorless to light brown and eventually dark brown under alkaline condition (Fig. 1) was the first sign of GTPs-capped AgNPs formation^32^. The cysteine was used as a linker that increases the efficiency and stability of nanoparticles (Fig. 1).

**Figure 3:**
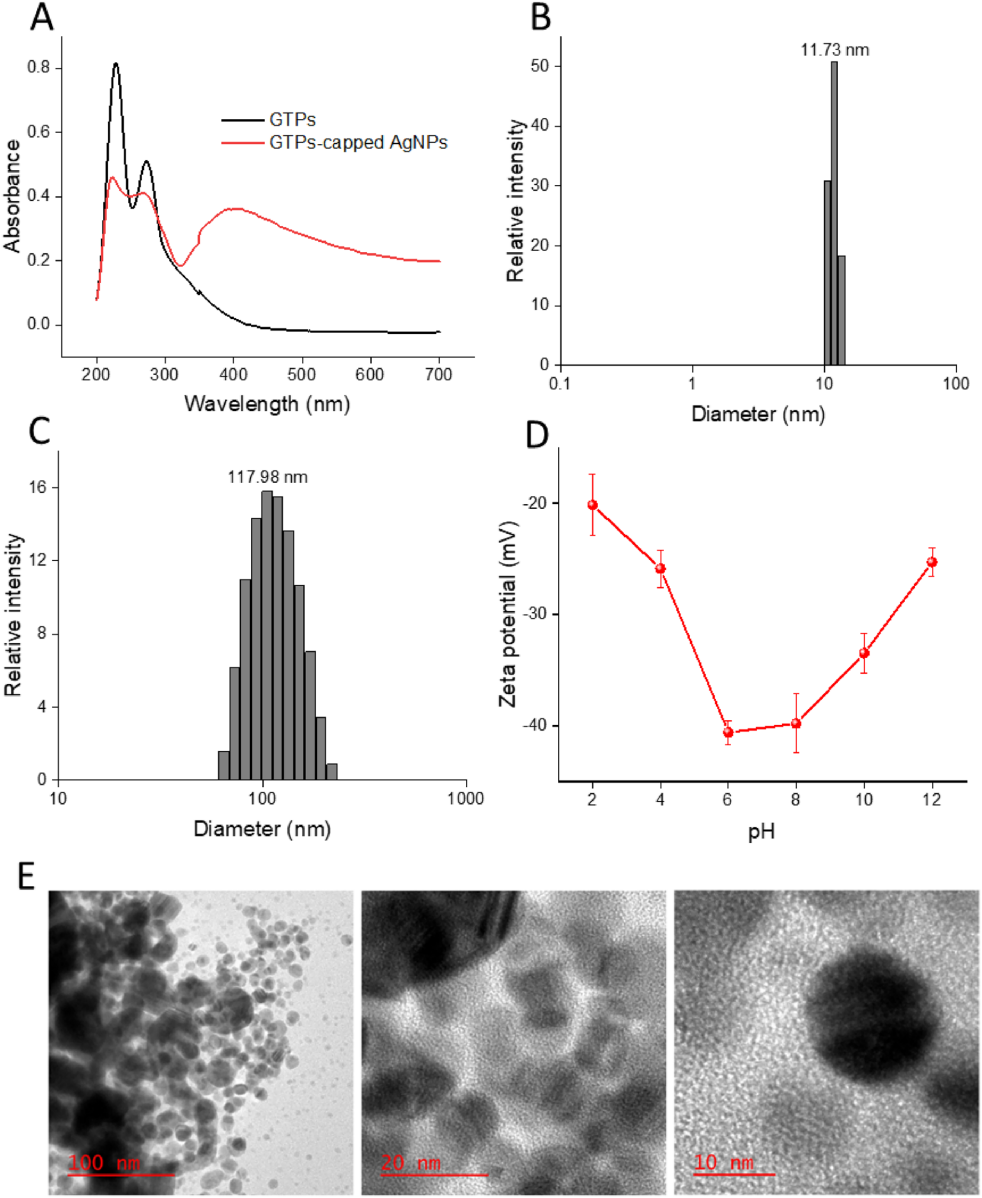
Spectroscopic and microscopic characterization of GTPs and GTPs-capped AgNPs. (**A**) UV–vis spectra of GTPs and GTPs-capped AgNPs aqueous solution (20 µg/mL). (**B**) Size distribution of GTPs and (**C**) GTPs-capped AgNPs. (**D**) Surface zeta potential of GTPs-capped AgNPs measured in deionized water. (**E**) HR-TEM images of GTPs-capped AgNPs indicate spherical structures with an average diameter of about 20 nm.

As depicted in Fig. 3A, the UV–Vis absorption spectra of GTPs shows two peaks at around 228 nm and 273 nm, characteristic of tea polyphenols including EGCG^33,34^. Upon nanoparticle formation, the intensity of peaks corresponding to GTPs decreased and a new peak at around 400 nm corresponding to silver nitrate appeared and intensified^35^, indicating the successful synthesis of GTPs-capped AgNPs. The synthesis of GTPs-capped AgNPs was further confirmed by size distribution measurements performed by dynamic light scattering (DLS) analysis. For the GTPs, we observed a narrow size distribution ranging from 10.51 to 13.41 nm with an average hydrodynamic size of 11.73 nm (related to some GTP aggregations) (Fig. 3B and Table S1). Upon nanonization, the size distribution pattern of GTPs-capped AgNPs shifted to large-sized species, ranging from 64.5 to 218.6 nm with an average hydrodynamic size of 117.98 nm (Fig. 3C and Table S1). The zeta potential measurement of GTPs-capped AgNPs showed negative zeta potential values in pH ranges of 2–12, where it decreased gradually with pH from − 20.12 to − 40.56 and then increased to − 25.28 mV (Fig. 3D).

This negative surface charge of GTPs-capped AgNPs may relate to the deprotonation of GTP hydroxyl groups. The zeta potential increasing from − 40.56 to − 25.28 mV by changing pH from 6 to 12 is likely attributed to the ion screening effect^36^. The high resolution-transmission electron microscopy (HR-TEM) micrographs revealed a spherical morphology for GTPs-capped AgNPs with an average diameter of about 20 nm (Fig. 3E)^32^. All these results confirm that the extraction of GTPs and synthesis of GTPs-capped AgNPs were successfully performed.

### GTPs-capped AgNPs exhibit higher antioxidant activity than GTPs

**Figure 4:**
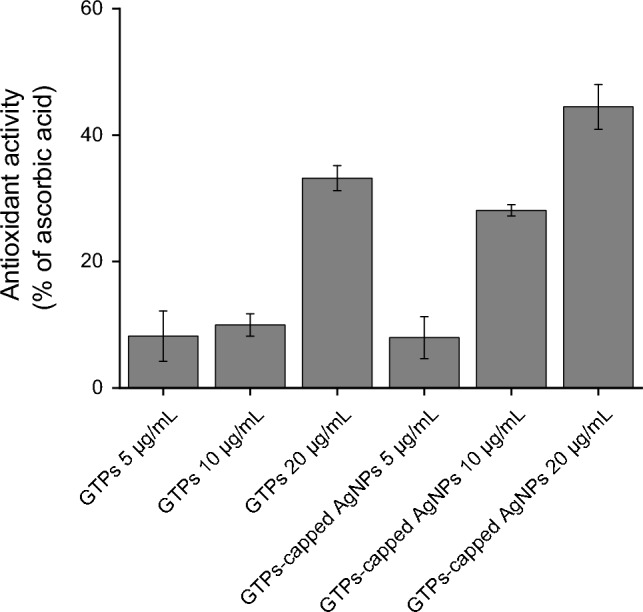
DPPH-based antioxidant activity of GTPs and GTPs-capped AgNPs. The results are calculated as a fraction of 20 µM ascorbic acid.

Since many neuroprotective properties of polyphenols, including inhibition of amyloid fibril formation, are attributed to their antioxidant activity, a DPPH-based antioxidant assay was employed to compare the capacity of GTPs and GTPs-capped AgNPs in scavenging DPPH^•^ free radicals^37^. While both GTPs and GTPs-AgNPs could scavenge the DPPH^•^ radicals in a concentration-dependent manner, GTPs-capped AgNPs were more efficient (Fig. 4).

A potential explanation for this observation could be attributed to the enhanced surface area of GTPs-capped AgNPs, which facilitates a more efficient interaction between the nanoparticles with DPPH^•^ radicals. In accordance with this observation, it has been shown that green synthesized metal nanoparticles exhibit higher antioxidant activity^38^. Given their enhanced antioxidant activities (Fig. 4) and minimal cytotoxicity (Figure S1), GTPs-capped AgNPs are well-suited for our intended use as antiamyloidogenic and neuroprotective compounds.

### GTPs-capped AgNPs are more efficient than GTPs in inhibiting amyloid fibril formation and disintegrating preformed amyloid fibrils

**Figure 5:**
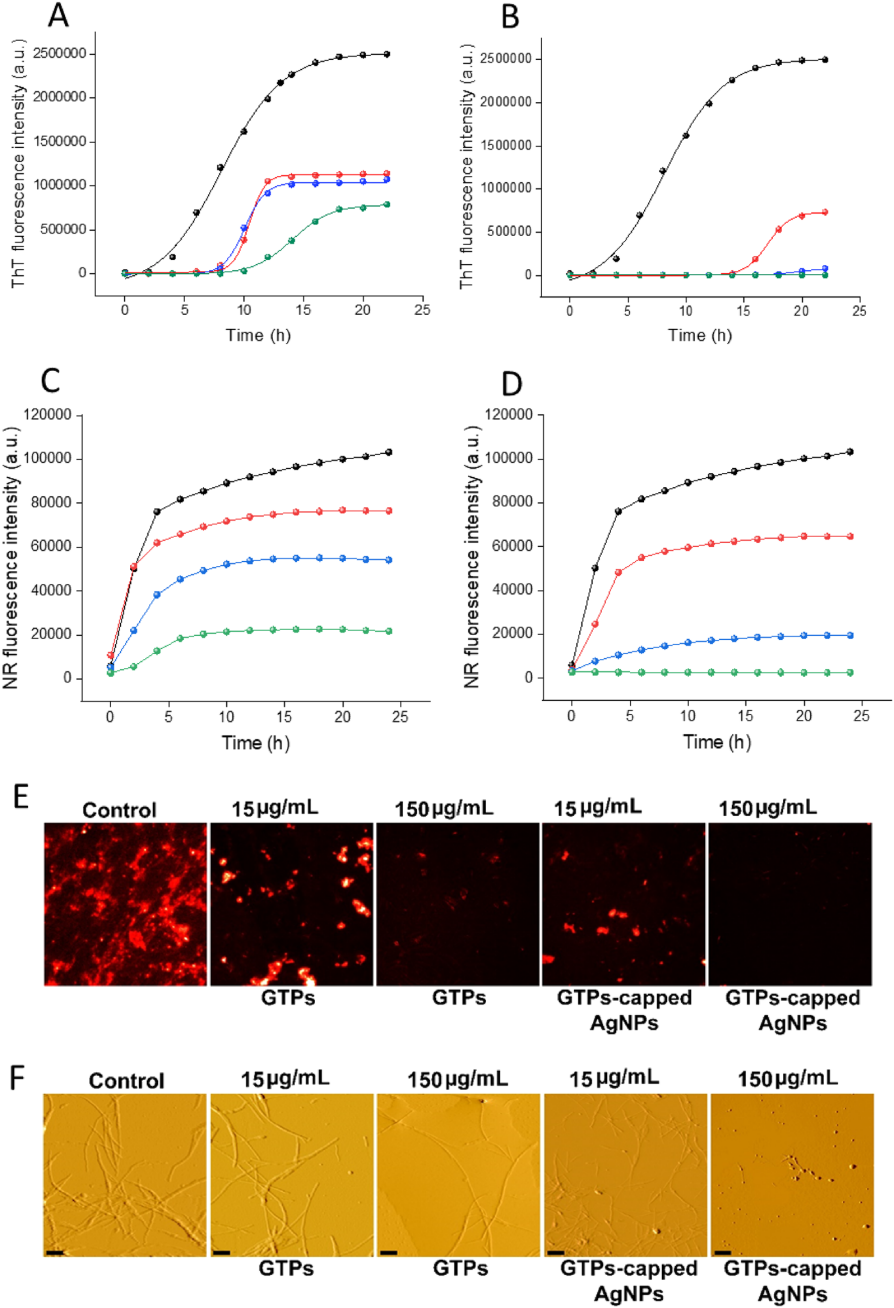
Effect of GTPs and GTPs-capped AgNPs on the amyloid fibrillation of human insulin. (**A** and **B**) Kinetics of human insulin fibrillation incubated without (black circle) or with 15 (red circle), 60 (blue circle), or 150 (green circle) µg/mL of GTPs or GTPs-capped AgNPs, respectively. (**C** and **D**) Changes in the surface hydrophobicity of human insulin incubated without (black circle) or with 15 (red circle), 60 (blue circle), or 150 (green circle) µg/mL of GTPs or GTPs-capped AgNPs, respectively. (**E** and **F**) Nile red fluorescence and AFM images of protein samples incubated either alone or with various concentrations of GTPs or GTPs-capped AgNPs. The scale bar represents 500 nm.

To investigate the capacity of GTPs and GTPs-capped AgNPs in inhibiting amyloid fibril formation as well as disintegrating preformed amyloid fibrils two proteins human insulin and α-syn were employed. The kinetics of amyloid fibril formation was monitored using ThT fluorescence assay. The results presented in Fig. 5A, B clearly show that GTPs-capped AgNPs are more efficient than GTPs in inhibiting amyloid fibril formation of human insulin. For example, while a dose-dependent increase in the nucleation phase was observed in samples incubated with either GTPs or GTPs-capped AgNPs, the extent of prolongation was significantly higher in samples containing GTPs-AgNPs (Fig. 5B). Based on these results, we suggest that the delay in primary nuclei formation, achieved through monomer stabilization, may be the mechanism by which GTPs, and particularly GTPs-capped AgNPs, modulate the assembly process of human insulin. To confirm this hypothesis, we investigated the conformational changes of incubated samples using Nile red (NR) spectroscopy and microscopy measurements. As shown in Fig. 5C, D, we observed a dose-dependent decrease in NR fluorescence intensity in samples treated with various concentrations of GTPs or GTPs-capped AgNPs. However, in protein solutions incubated with GTPs-capped AgNPs, the extent of inhibition was pronounced. For instance, in samples containing 50 µg/mL GTPs-capped AgNPs, there was no detectible increase in the fluorescence intensity of NR (Fig. 5D).

**Figure 6:**
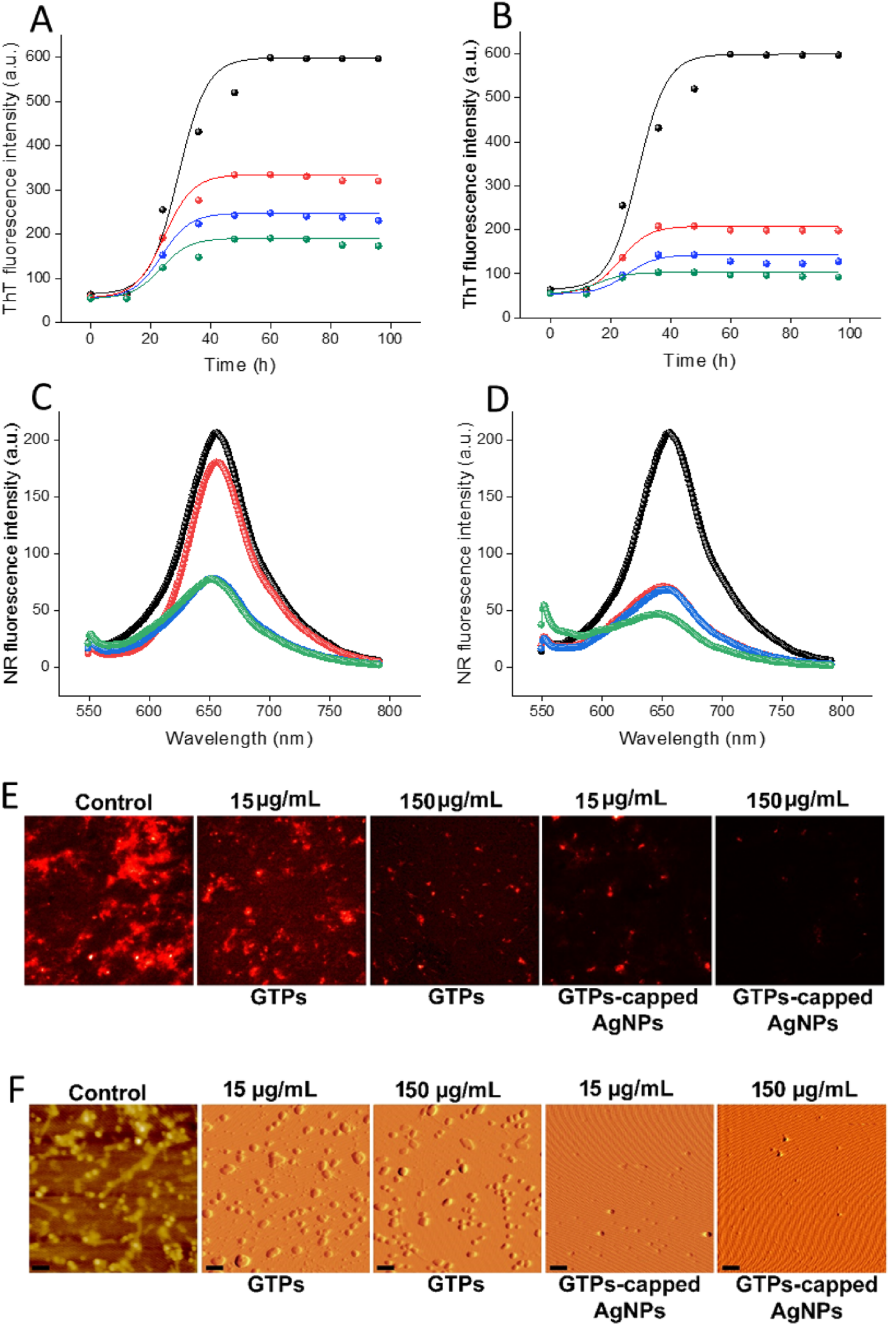
Effect of GTPs and GTPs-capped AgNPs on the amyloid fibrillation of α-syn. (**A** and **B**) Kinetics of α-syn fibrillation incubated without (black circle) or with 15 (red circle), 75 (blue circle), or 150 (green circle) µg/mL of GTPs or GTPs-capped AgNPs, respectively. (**C** and **D**) Changes in the surface hydrophobicity of α-syn incubated without (black circle) or with 15 (red circle), 75 (blue circle), or 150 (green circle) µg/mL of GTPs or GTPs-capped AgNPs, respectively. (E and F) Nile red fluorescence and AFM images of protein samples incubated either alone or with various concentrations of GTPs or GTPs-capped AgNPs. The scale bar represents 300 nm for untreated and 500 nm for treated samples, respectively.

Fluorescence microscopy further confirmed these results, indicating that GTPs-capped AgNPs exhibit higher performance in impeding the exposure of hydrophobic patches during fibril formation (Fig. 5E). According to these data, we suggest that GTPs-capped AgNPs, by providing a larger surface area, are more effective in inhibiting the conformational changes required for amyloid fibril formation^39–41^. This improved efficiency of GTPs-capped AgNPs was further confirmed by Congo red (CR) binding assay, which served as a complementary evaluation of amyloid fibril formation. While both GTPs and GTPs-capped AgNPs effectively inhibited the enhancement in CR absorbance, prevention of the red shift was only observed in samples containing GTPs-capped AgNPs (Figure S2), further supporting the ThT and NR fluorescence data (Fig. 5A–E). Finally, for the morphological characterization of incubated samples, we employed AFM. While both GTPs and GTPs-capped AgNPs could dose-dependently inhibit the formation of human amyloid fibrils, GTPs-capped AgNPs exhibited higher efficiency. As illustrated in Fig. 5F, in the sample containing 50 µg/mL GTPs-capped AgNPs the formation of amyloid fibrils was completely inhibited and instead, some non-amyloid spherical structures were observed. The AFM results were further confirmed by ThT fluorescence microscopy (Figure S3). To investigate the possible interaction of GTPs/GTPs-capped AgNPs with nuclei/small oligomers formed at the early stages of fibril formation, we performed seeding experiments with sonicated seeds derived from human insulin fibrils. In the control samples, adding the seeds (2.5% v/v) reduced the lag time of human insulin fibrillation (Figure S4). We attribute this exponential growth of amyloid fibrils to secondary nucleation at the surface of provided seeds^42,43^. In the presence of GTPs, there was a slight decrease in ThT fluorescence, indicative of some inhibition of human insulin fibril formation (Figure S4A). In contrast, in the case of samples containing GTPs-capped AgNPs, the seeding effect was substantially inhibited in a dose-dependent manner, as we did not observe any detectable emission for samples containing the highest concentration of GTPs-capped AgNPs (Figure S4B). This finding suggests that the interaction of added seeds with amyloidogenic species has been inhibited in the presence of GTPs-capped AgNPs, likely due to their higher surface area, which promotes more efficient binding of these nanoparticles to the protein and consequently suppresses the secondary pathways during fibrillation process. To determine whether the anti-amyloidogenic activity of GTPs/GTPs-capped AgNPs is specific to human insulin or has broader implication for other disease-related proteins, we investigated the effectiveness of these compounds in inhibiting the amyloidogenesis of α-syn, a protein associated with Parkinson’s disease. The data obtained by ThT and NR fluorescence measurements, CR binding assay, and fluorescence and atomic force microscopies (Fig. 6 and Figure S5) clearly demonstrate that GTPs-capped AgNPs are more efficient in preventing the assembly process of α-syn compared to GTPs alone. This indicates that the anti-amyloidogenic activity of GTPs/GTPs-capped AgNPs extends beyond human insulin and may have potential applications for other disease-related proteins. However, the mechanism by which GTPs/GTPs-capped AgNPs modulate fibril formation of α-syn appears completely different from that for the human insulin. For example, a decrease in the intensity of ThT fluorescence in samples incubated with either GTPs or GTPs-capped AgNPs was observed without any significant prolongation of the nucleation phase (Fig. 6A, B). Moreover, AFM images indicate redirecting of the α-syn aggregation pathway to the formation of amorphous aggregates in samples containing GTPs. In the case of GTPs-capped AgNPs, the extent of inhibition was more effective so that some small oligomers were observed in the presence of 150 µg/mL of these nanoparticles (Fig. 6F). These results further confirm previous reports demonstrating that the assembly process of natively unfolded polypeptides, including α-syn, can be redirected into amorphous aggregates or off-pathways oligomers by the polyphenol EGCG, as the main polyphenolic compound of green tea^49^.

For the structured protein human insulin, however, we observed prolongation of the nucleation phase with the appearance of short fibrils and spherical oligomers without forming amorphous aggregates (Fig. 5F). These data may suggest that the mechanism by which these compounds exert their anti-amyloidogenic effects may be specific to the target proteins. Interestingly, EGCG has been shown to inhibit the assembly process of different proteins/peptides through different mechanisms^44,45^. The molecular mechanisms underlying the amyloid assembly process of ordered and intrinsically disordered proteins are different^46^. While fibrillation of ordered proteins such as human insulin involves structural destabilization and formation of partially unfolded conformation, for intrinsically disordered proteins like α-syn to fibrillate, their flexible and unfolded structure has to be partially folded rather than unfolding^46^. This means that, at least in part, fibrillation inhibition of human insulin requires constraining of monomer in its native state. On the other hand, to inhibit fibrillogenesis of α-syn, GTPs/GTPs-capped AgNPs have to prevent stabilizing a partially folded beta-rich conformation. Taken together, these data suggest that while inhibition of amyloid fibril formation is a generic feature of GTPs, and particularly GTPs-capped AgNPs, the mechanism of action of these compounds may be dependent on the sequence of target protein. The ability of GTPs/GTP-capped AgNPs to disintegrate preformed fibrils of proteins was investigated by ThT fluorescence assay. For both proteins we observed a similar trend; the efficient disintegrating of human insulin and α-syn mature fibrils with higher potency of GTPs-capped AgNPs compared to GTPs (Fig. S6). Increased intensity of ThT fluorescence in control samples indicates that the formation of amyloid fibrils has continued under this condition (Fig. S6). Taken together, two major conclusions may be derived from these results. First, GTPs, and particularly GTPs-capped AgNPs, are efficient inhibitors for both human insulin and α-syn fibril formation and disintegrating preformed fibrils. However, the mode by which these molecules exert their anti-aggregation activities may vary depending on target peptides/proteins. Second, the higher anti-amyloidogenic activity of GTPs-capped AgNPs, compared to their respective molecular form, may be attributed to their increased surface area according to the surface-assistance model^47–49^, leading to more efficient interaction with protein species of nanoparticles. This means that GTPs-capped AgNPs may deplete a large fraction of monomeric proteins from the solution resulting in a decreased aggregation rate.

### GTPs-capped AgNPs are more efficient than GTPs in inhibiting cytotoxicity and hemolysis induced by amyloid fibrils

**Figure 7:**
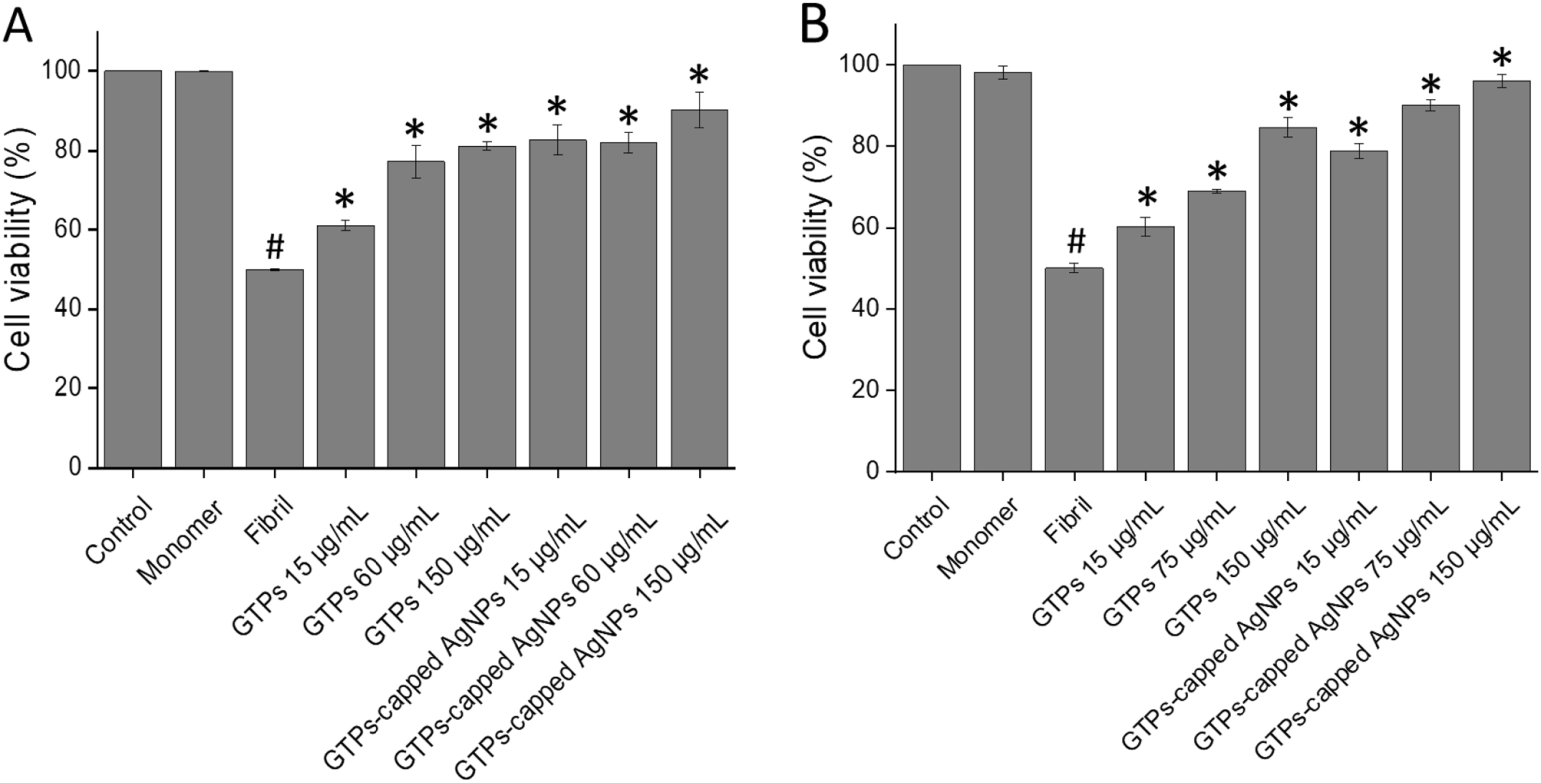
Cytotoxicity evaluation of (**A**) human insulin and (**B**) α-syn aggregates produced in the absence or presence of increasing concentrations of GTPs or GTPs-capped AgNPs. Further descriptions are provided in the text. ^#^*p* < 0.01, significantly different from control cells. ^*^p < 0.01, significantly different from cells exposed only to amyloid fibrils.

Next, we investigated the performance of GTPs/GTPs-capped AgNPs in lowering cytotoxicity induced by the amyloid aggregates arising from human insulin and α-syn. This was performed using human neuroblastoma SH-SY5Y cell line and evaluated by conventional MTT assay, intracellular ROS and mitochondrial membrane potential measurements. Based on our previous reports, 20 µM amyloid fibrils, as the concentration causing about 50% decrease in cell survival, was used for subsequent toxicity experiments. As depicted in Fig. 7A, cytotoxicity of human insulin amyloid fibrils was remarkably prevented by the presence of either GTPs or GTPs-capped AgNPs in a concentration-dependent manner. Similar results regarding cytotoxicity induced by α-syn aggregates (Fig. 7B) were obtained. In both incubated samples, however, the efficiency of GTPs-capped AgNPs in reducing the amyloidogenic toxicity was more significant than the molecular GTPs. As shown in Fig. 7A, B, the low concentrations of GTPs-capped AgNPs increased cell survival to levels similar to those exerted by the high concentrations of molecular GTPs.

**Figure 8:**
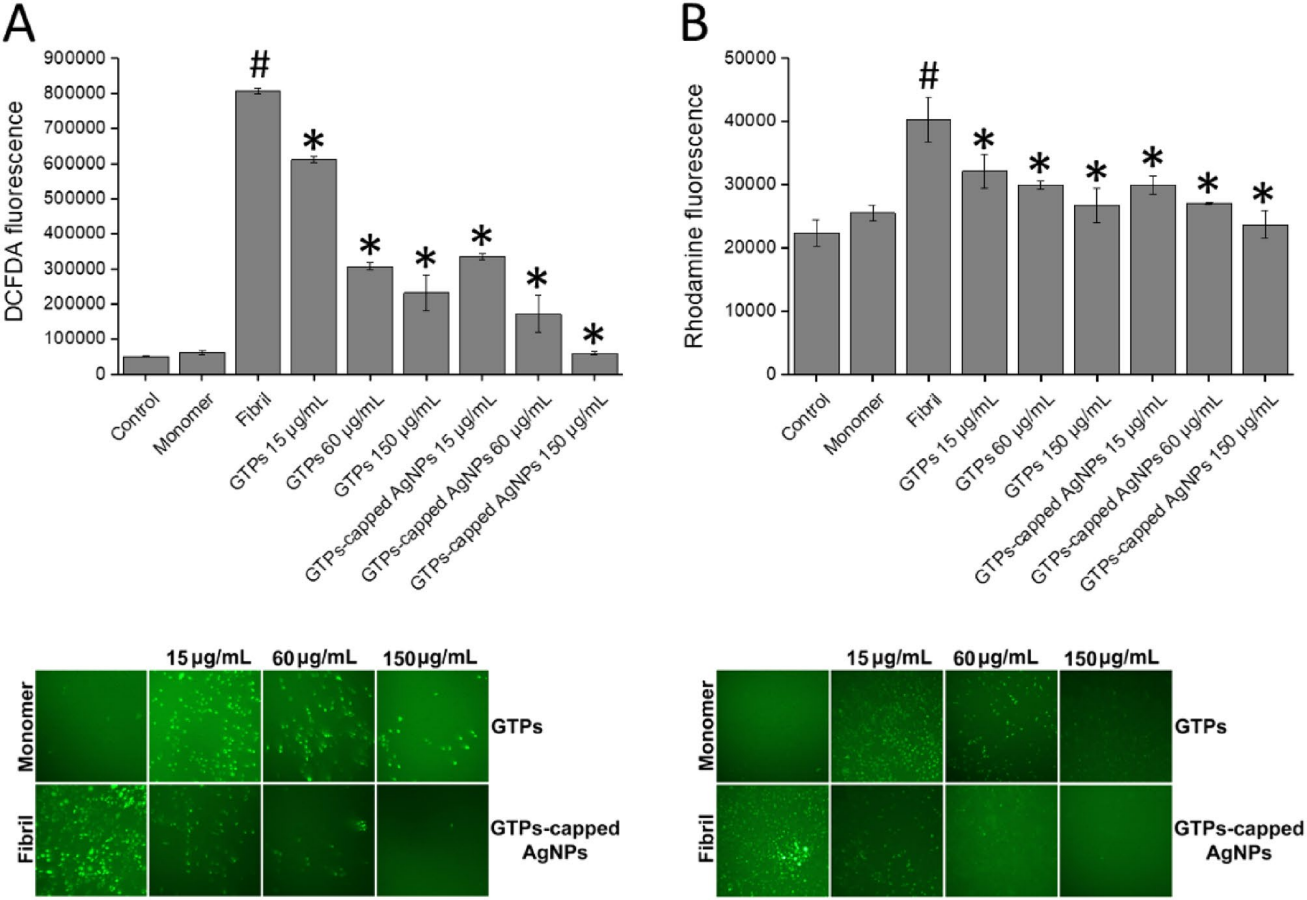
Effect of GTPs and GTPs-capped AgNPs on the human insulin amyloid fibrils-induced (**A**) intracellular ROS content and (**B**) mitochondrial membrane potential in SH-SY5Y cells evaluated by DCFDA and Rhodamine 123 fluorescence assays, respectively. Further details are described in the text. Low panels show fluorescence microscopy images of incubated samples. ^#^*p* < 0.01, significantly different from control cells. ^*^p < 0.01, significantly different from cells exposed only to amyloid fibrils.

**Figure 9:**
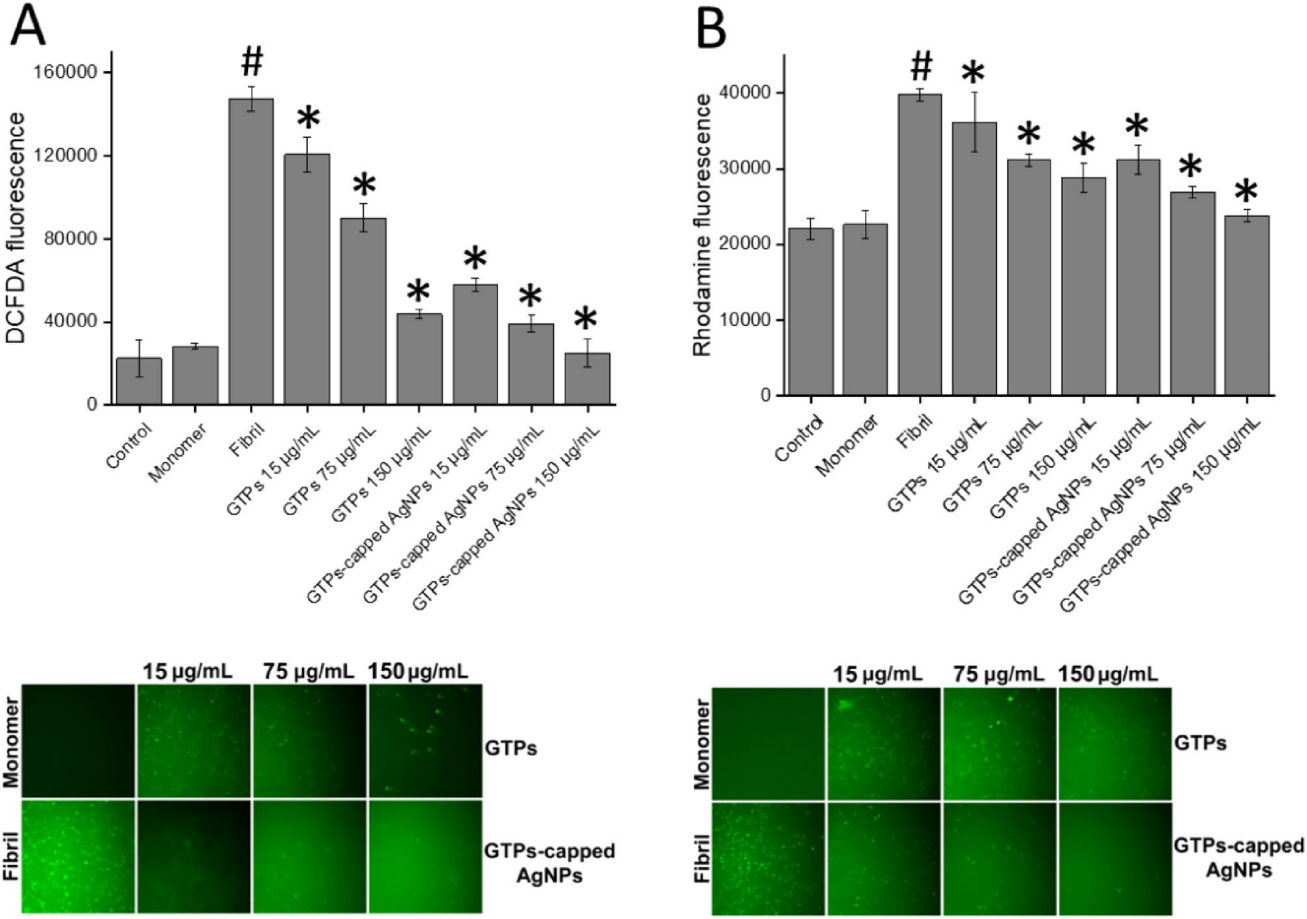
Effect of GTPs and GTPs-capped AgNPs on the α-syn amyloid fibrils-induced (**A**) intracellular ROS content and (**B**) mitochondrial membrane potential in SH-SY5Y cells evaluated by DCFDA and Rhodamine 123 fluorescence assays, respectively. Further details are described in the text. Low panels show fluorescence microscopy images of incubated samples. ^#^*p* < 0.01, significantly different from control cells. ^*^p < 0.01, significantly different from cells exposed only to amyloid fibrils.

This data may indicate that protein species produced in the presence of GTPs-capped AgNPs have reduced efficiency to interact with cell components, including membranes. A possible explanation for this observation may be related to the more effective interaction of GTPs-capped AgNPs, compared to their respective molecular form, with hydrophobic patches exposed under amyloidogenic conditions, leading to a substantial decrease in their surface hydrophobicity (Figs. 5D and 6D), and consequently, their decreased performance to interact with and disrupt membrane integrity. This conclusion is in accordance with previous reports indicating the potency of polyphenols to bind solvent-exposed hydrophobic patches of proteins^50–52^. The potency of GTPs/GTPs-capped AgNPs to decrease cytotoxicity associated with human insulin or α-syn aggregates was further examined by measuring intracellular ROS content and mitochondrial membrane potential, and microscopic imaging. Based on the results presented in Figs. 8and 9, treatment of SH-SY5Y cells with 20 µM amyloid fibrils led to a significant enhancement in intracellular ROS content and a considerable loss of mitochondrial membrane potential, in accordance with previous reports^53,54^.

These destructive effects of amyloid aggregates may be attributed to the progressive impairment of mitochondrial function and increased ROS production as one of the mechanisms involved in amyloid aggregates toxicity in relation to neurodegenerative diseases^55,56^. This increased ROS production can exacerbate mitochondrial dysfunction and promote oxidative damage to mitochondrial phospholipids, particularly cardiolipin, leading to the release of proapoptotic agents such cytochrome c, thereby inducing cell death pathways including mitochondria-mediated apoptosis^57^. The presence of GTPs, and specifically GTPs-capped AgNPs, significantly and dose-dependently counteracted these toxic effects of protein aggregates (Figs. 8 and 9).

**Figure 10:**
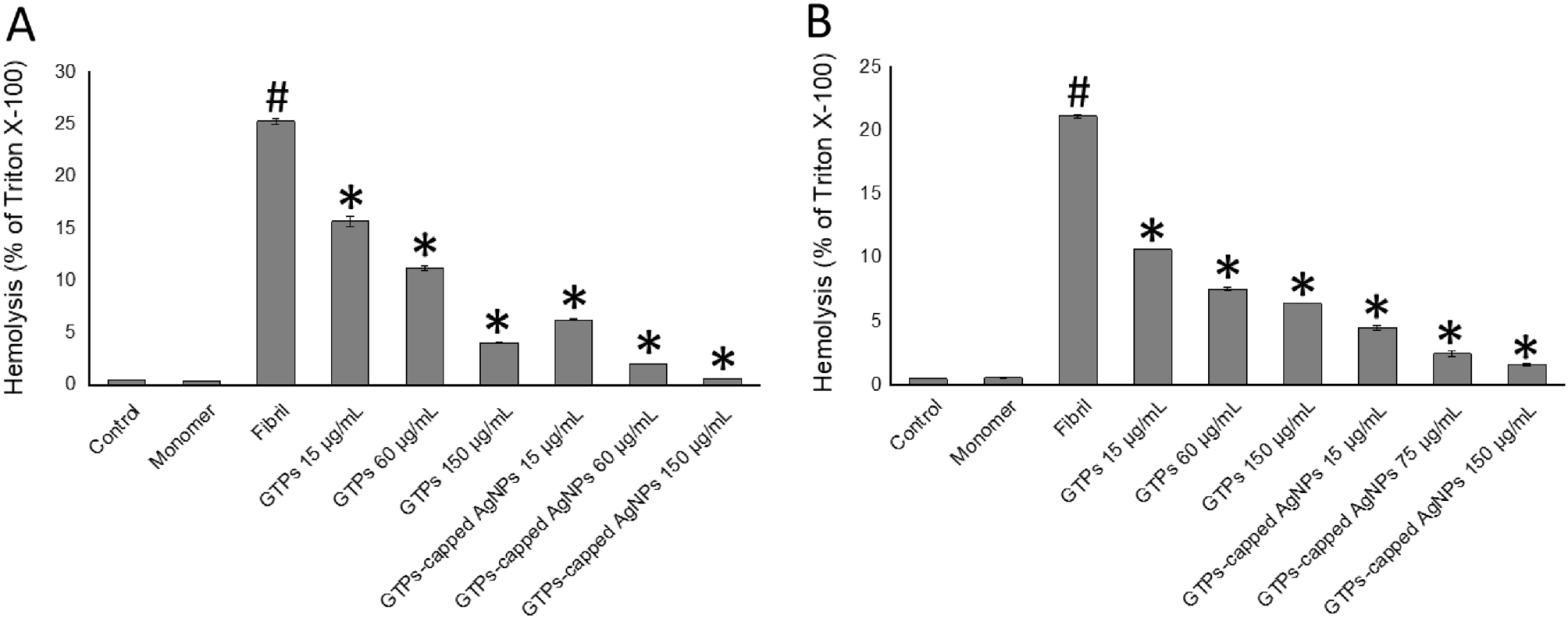
Effect of the GTPs and GTPs-capped AgNPs on the hemolytic activity of (**A**) human insulin and (**B**) α-syn amyloid fibrils. The percentage of hemolysis is calculated as a fraction of Triton X-100. ^#^*p* < 0.01, significantly different from control cells. ^*^p < 0.01, significantly different from cells exposed only to amyloid fibrils.

Finally, the improved protective effects of nanoparticles were further corroborated by investigating their capacity to protect erythrocyte membranes against permeabilization induced by human insulin or α-syn amyloid fibrils. As shown in Fig. 10, while monomers of human insulin and α-syn were ineffective, treatment of erythrocytes with 20 µM amyloid fibrils resulted in a significant membrane permeabilization.

In the presence of either GTPs or GTPs-capped AgNPs, however, a dose-dependent decrease in hemolysis rate was observed, suggesting that structures produced in the presence of these components have lost their membrane permeabilization activity. Moreover, at the same concentrations, GTPs-capped AgNPs were more effective than molecular GTP in protecting erythrocytes membranes (Fig. 10), which is in accordance with cell toxicity results (Figs. 7, 8 and 9). While preventing structural/conformational changes, which are necessary for effective interaction and binding of amyloid aggregates to cell membranes, may be involved in decreasing amyloidogenic toxicity induced by molecular and nano forms of GTPs, but other mechanisms may be considered. Many reports indicate the potency of natural polyphenols with antioxidant properties, including GTPs, in attenuating oxidative damage and protecting cell components against toxicity induced by amyloid aggregates^58,59^. Accordingly, the higher antioxidant activity of GTPs-capped AgNPs, compared to their molecular form (Fig. 4), may provide these nanoparticles with increased capacity to counteract oxidative damage and protect mitochondrial function (Figs. 8 and 9). To further confirm improved mitochondrial protective effects of GTPs-capped AgNPs, we must perform other experiments, including intracellular ATP content measurement, mitochondrial permeability transition, and mitochondrial calcium content determination. Another factor that appears to be involved in increased anti-amyloidogenic and neuroprotective effects of GTPs-capped AgNPs is the higher surface/volume ratio. In fact, thanks to their increased surface/volume ratio and in agreement with the surface-assistant model^47–49^, GTPs-capped AgNPs would be able to absorb amyloidogenic species more effectively than their respective molecular form, and thereby decrease solution concentration and aggregation rate of proteins.

## Conclusion

**Figure 11:**
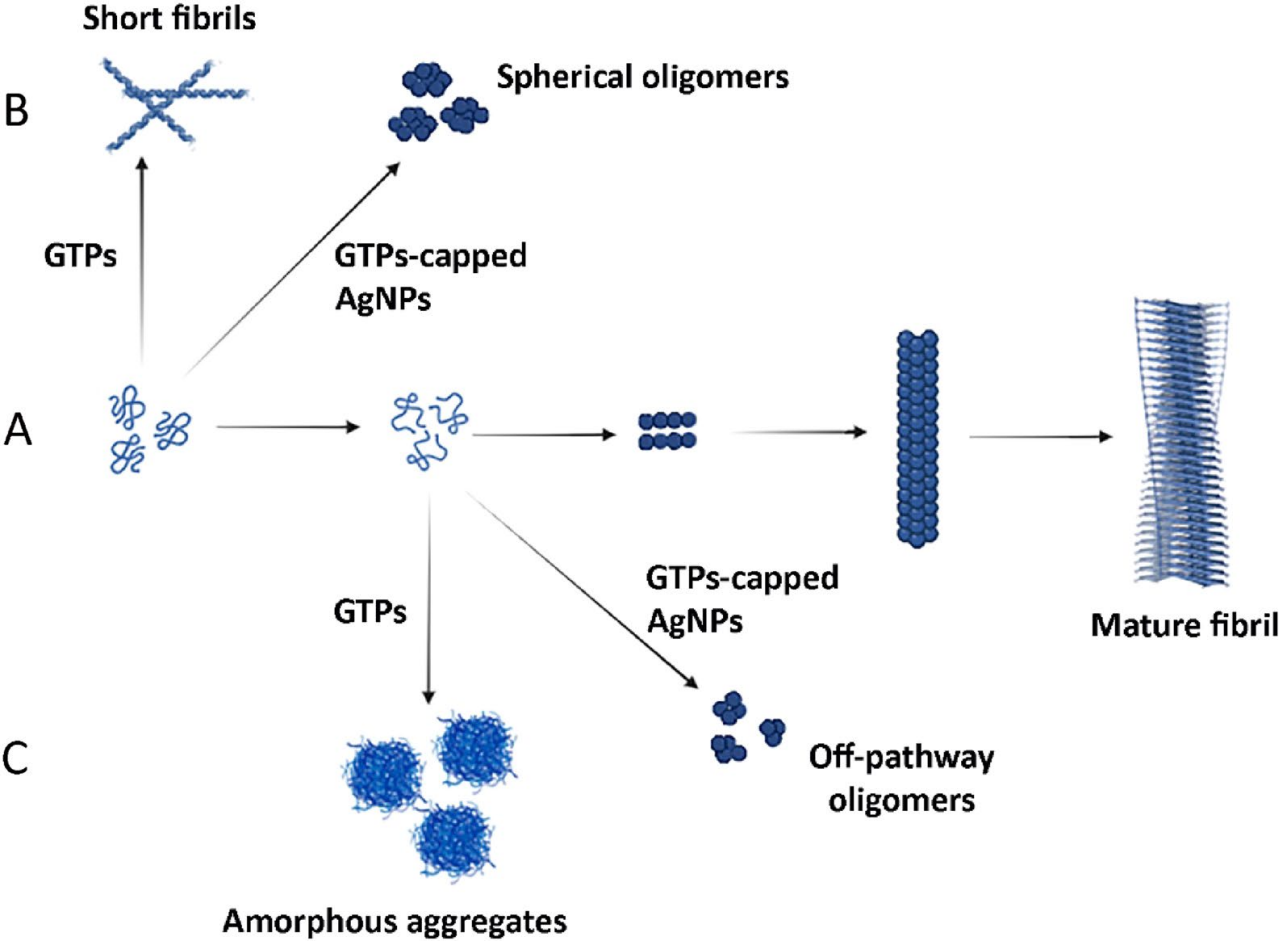
Schematic representation of possible mechanisms by which GTPs/GTPs-capped AgNPs modulate aggregation pathway of proteins. (**A**) The pathway of amyloid fibril formation starting with monomers that assemble to form on-pathway oligomers growing up to mature amyloid fibrils. (**B**) For the structured protein human insulin, the presence of GTPs or GTPs-capped AgNPs alters the amyloid assembly pathway toward the formation of short fibrils or spherical oligomers, respectively. (**C**) For the natively unfolded protein α-syn, the presence of GTPs or GTPs-capped AgNPs alters the amyloid assembly pathway toward the formation of amorphous aggregates or off-pathways oligomers, respectively. Molecule templates were retrieved from BioRender databases.

In summary, our findings indicate that GTPs-capped AgNPs are more effective than their respective molecular form in inhibiting protein aggregation and reducing the cytotoxicity of amyloid fibrils derived from human insulin and α-syn. We believe this increased efficacy of GTPs-capped AgNPs is likely due to their enhanced surface/volume ratio. In fact, with a large surface area, these nanoparticles can interact more effectively with surrounding molecules, including monomeric proteins and reactive species. As a result, the anti-amyloidogenic and antioxidant activities of these nanoparticles are improved, leading to better protection against toxicity induced by amyloid fibrils. Although both molecular, and particularly nano, forms of GTPs are effective inhibitors of human insulin and α-syn amyloid fibrillation, our findings suggest that their mechanisms of action may differ and depend on the target peptide/protein, which is in agreement with previous reports^44,45^. For structured human insulin, GTPs-capped AgNPs bind more effectively to monomers and thereby stabilize native proteins leading to inhibition of formation of partially unfolded conformation which is necessary for amyloid fibril formation. These interactions redirect the assembly process of protein toward the formation of non-toxic spherical oligomers. For intrinsically disordered α-synuclein protein, GTPs-capped AgNPs are suggested to bind to partially unfolded species and prevent their conversion into toxic, on-pathway aggregation intermediates. Instead, GTPs-capped AgNPs promote the fibrillation process toward the formation of non-toxic off-pathway oligomers (Fig. 11). Furthermore, the protein/polyphenol molar ratios used in this study are remarkably lower than those reported in previous works^44,60^. A possible explanation for this may be related to the synergistic effects of polyphenolic extracts, which can improve their anti-amyloidogenic and neuroprotective properties^61,62^.

Our preliminary experiments indicate significant fluorescence emission from GTPs-capped AgNPs and their potency to bind and traverse biological membranes (data not shown), which further supports their potential as molecular probes for detection of intracellular protein aggregates. Although the results are robust and promising, further in vitro and in vivo experiments are necessary to validate these findings and gain a better understanding of the molecular mechanisms underlying the action of these nanoparticles in various model systems.

## Materials and methods

### Extraction of GTPs and synthesis of GTPs-capped AgNPs

Iranian green tea was prepared from the market in Rasht, Iran. Three grams of green tea were finely powdered, added to 50 mL of deionized water, and then heated at 65 °C for 10 min. The mixture was centrifuged at 9000 rpm for 20 min. The supernatant, named green tea polyphenols (GTPs), was collected, dried in an oven, and stored at 4 °C for subsequent experiments. To synthesize GTPs-capped AgNPs, 1 mL of cysteine solution (2 mM) was added to 99 mL of silver nitrate solution (1 mM) and mixed for 10 min at room temperature. Then, 5 mL of GTPs (5 mg/mL) were slowly added to the solution while being stirred. A color changing from colorless (before GTPs addition) to light-brown (after GTPs addition) and then to dark brown (after adjusting pH to 11, and heating at 65 °C for 30 min) indicates the formation of GTPs-capped AgNPs (Fig. 1). The obtained nanoparticles were centrifuged at 20,000 rpm for 30 min at room temperature, and the resultant pellet was collected and dried in an oven at 37 °C. The obtained powder was finely ground and stored in a dark place for subsequent experiments.

### Characterization of GTPs and GTPs-capped AgNPs

First, LC–MS was employed to examine the constituents of GTPs. The analysis was performed using a Shimadzu LC–MS 2010 A, equipped with an Eclipse Atlantis T3 column (100 mm × 2.1 mm × 3 µm particle size) (Waters, USA) with an electrospray ionization ESI source. The binary gradient of 1% v/v formic acid in deionized water (solvent A) and acetonitrile (solvent B) at a constant flow rate of 0.2 mL/min was applied with an injection volume of 5.0 µL. The column was operated at room temperature. The detection gain was 1.8 kV, and probe and CDL voltages were 3.5 kV and 20 V, respectively. Grade 5 nitrogen gas was employed as the nebulizer with a flow rate of 1.2 L/min. The CDL and block temperatures were both 250 °C. The data was collected by the Lab Solutions TM software. The UV–Vis spectrum of GTPs and GTPs-capped AgNPs at a final concentration of 20 μg/mL was recorded using a spectrophotometer (UV-2550, Shimadzu). DLS experiments were carried out using a zeta potential and particle size analyzer (HORIBA Scientific, SZ100, Japan). For size distribution measurement, aliquots of GTPs and GTPs-capped AgNPs (at a final concentration of 10 µg/mL) were illuminated by a laser of 532 nm with a fixed detector angle of 90° at 25 °C. For surface zeta potential measurement, samples were diluted with deionized water to a final concentration of 20 µg/mL. Each experiment was performed at least five times and the average was used for data analysis. Finally, to monitor the morphology of nanoparticles, aliquots of an aqueous suspension of GTPs-capped AgNPs (20 µg/mL) were placed on a carbon-coated copper grid and blotted after 30 s. The images were acquired with HRTEM (JOEL JEM 2010).

### DPPH radical scavenging activity

Free radical scavenging capacity of samples was determined based on the DPPH assay as a standard method for quantitating antioxidant content^37^. Twenty-five µL of GTPs, GTPs-capped AgNPs, or deionized water as control was added to 475 µL of methanolic DPPH solution (25 µM) such that the final concentrations of the components were in the range of 5 to 20 μg/mL. Ascorbic acid (20 µΜ) was used as positive control. The reaction mixtures were transferred into a polystyrene 96-well plate, and the absorbance values of samples were recorded at 517 nm after 30 min using a microplate reader (BioTek Instruments, Winooski, VT 05,404-0998, USA). All experiments were performed in triplicate. The percentage inhibition of the free radical scavenging activity of compounds was expressed as the fraction of maximum effect (ascorbic acid): [(measured signal − blank signal)/(maximum signal − blank signal)] × 100.

### Sample preparation and amyloid fibril formation

Human insulin was dissolved in 50 mM glycine buffer (pH 2.2) in a final concentration of 1.5 mg/mL (∼200 µΜ). Recombinant human α-syn was expressed on Escherichia coli BL21 containing plasmid PT7-7 (Addgene) encoding for the protein. The expressed protein was purified as described previously^63–65^. The protein was aliquoted and stored at − 20 °C without any preservative. Dry powders of GTPs and GTPs-capped AgNPs were dissolved in deionized water at a final concentration of 50 mg/mL. All stocks were stored at − 20 °C until use. For human insulin amyloid fibrillation, aliquots of proteins (100 µL), containing 20 µΜ thioflavin T (ThT) and various concentrations of GTPs or GTPs-capped AgNPs (0, 15, 60, 150 μg/mL), were transferred into a nonbinding black surface with a transparent bottom polystyrene 96-well plate (Corning Incorporated, 2 Alfred Road, Kennebunk ME 04,043 USA). The plate was sealed with a crystal-clear sealing tape (Axygen, Corning Incorporated USA) and loaded into a Synergy Hybrid Multi-Mode microplate reader (BioTek Instruments, Winooski, VT 05,404-0998, USA) followed by incubation at 57 °C with agitation. The fluorescence signal was recorded at 20 min intervals, with excitation at 440 nm and emission at 485 nm for 22 h. For α-syn fibril formation, the protein was dissolved in phosphate buffered saline (PBS) to a final concentration of 1.5 mg/mL (∼ 100 µΜ). The aliquots, containing 20 µΜ ThT, were incubated at 37 °C (without or with 15, 75, or 150 μg/mL GTPs or GTPs-capped AgNPs) under constant stirring at 1000 rpm for 4 days. To investigate the effect of GTPs/GTPs-capped AgNPs on the secondary nucleation of fibrillation process seeding experiment was conducted. Briefly, preformed amyloid fibrils of human insulin were centrifuged at 15,000 × g for 10 min to eliminate prefibrillar aggregates. To prepare the seeds, amyloid fibrils were sonicated for 3 min using a probe sonicator. The seeds (2.5% v/v) were added to protein solutions containing 20 µM ThT and increasing concentrations of GTPs or GTPs-capped AgNPs at a final volume of 100 µL. The protein solutions of human insulin were transferred in triplicates to a black 96-well plate with a transparent bottom. The plate was then sealed with Crystal Clear sealing tape. The samples were incubated at 57 °C, while being shaken at 1000 rpm. To examine the effects of GTPs/GTPs-capped AgNPs on the conformational changes of protein samples under amyloidogenic conditions, NR fluorescence measurements were conducted. For human insulin, 100 µL of protein at a concentration of 1.5 mg/mL were prepared. These aliquots contained 20 µM NR and various concentrations of GTPs or GTPs-capped AgNPs (0, 15, 60, 150 μg/mL). The aliquots were transferred to a nonbinding black surface with a clear bottom polystyrene 96-well plate. Following this, the plate was incubated under the conditions described above. The fluorescence signal was recorded at 2 h intervals for a duration of 24 h, with excitation at 530 nm and emission at 650 nm. For α-syn, protein aliquots were removed at the end of incubation period. These aliquots were then diluted to a final concentration of 2 µM in PBS (pH 7.4) containing 20 µΜ NR. Samples were excited at 530 nm, and emission spectra were recorded from 540 to 800 nm. To further confirm NR results, 10 µL of incubated human insulin and α-syn proteins were diluted with glycine buffer or PBS, respectively, to a final concentration of 10 µΜ and incubated for 10 min in a dark place at room temperature. The mixtures were placed on clean glass slides and air-dried. The images were captured using a fluorescence microscope (Zeiss, Germany) at 20 × magnification. For CR binding assay, incubated aliquots of human insulin or α-syn (at a final concentration of 2.5 µΜ) solutions were added to 950 µL of CR solution (20 µΜ). After 30 min of incubation at room temperature, absorbance spectra were recorded between 400 and 600 nm. Finally, for AFM imaging, aliquots of incubated human insulin or α-syn solutions were removed and diluted with deionized water to a final concentration of 5 µΜ. Then, 10 µL of diluted sample was placed on a freshly cleaved mica and dried at room temperature. Images were acquired in non-contact mode using a quantitative AFM (ARA-AFM, Ara-Research Company, Iran). To evaluate the potency of GTPs/GTPs-capped AgNPs in disintegration preformed amyloid fibrils, matured fibrils of bovine insulin and α-syn were prepared by incubation of protein solutions under amyloidogenic conditions for sufficient time, as described before. Aliquots (100 μL) of preformed amyloid fibrils were then incubated with increasing concentrations of GTPs or GTPs-capped AgNPs at 37 °C for 20 h followed by measuring changes on the intensity of ThT fluorescence.

### Cell toxicity assays

Human neuroblastoma SH-SY5Y cells were cultured in DMEM medium, supplemented with 10% fetal bovine serum, streptomycin (100 μg/mL), and penicillin (100 U/mL), and kept at 37 °C in a 5% CO_2_ humidified atmosphere. Cells were seeded in a 96-well plate at a density of 2 × 10^4^ cells/well, and the medium was changed before incubation with protein aggregates. To evaluate the involvement of the anti-amyloidogenic activity of compounds against toxicity induced by human insulin or α-syn amyloid fibrils, aliquots of protein samples (final concentration of 20 µΜ) aged without or with various concentrations of GTPs/GTPs-capped AgNPs under amyloidogenic conditions were added to the cells and left for 24 h. Cells treated with 50 mM glycine buffer or PBS were used as control. Cell viability was assessed using the conventional MTT reduction assay. Briefly, after treatment, the medium was replaced with 20 µL/well of MTT stock solutions (2 mg/mL in PBS), followed by incubation at 37 °C for 3 h. Solutions were aspirated, and cells were treated with 100 µL/well DMSO for 30 min, followed by absorbance reading at 570 nm on a microplate reader (BioTek). Results were expressed as a percentage of MTT reduction relative to the control cells, assuming that the absorbance of control cells was 100%. The mitochondrial ROS content was measured using the oxidation-sensitive fluorescent probe DCFDA^66^. Briefly, cells were seeded in a 96-well plate at 1 × 10^4^ cells/well density and incubated overnight. Then, SH-SY5Y cells were treated with 20 µΜ incubated samples aged alone or in the presence of various concentrations of GTPs/GTPs-capped AgNPs and incubated for 24 h. The medium was replaced with 100 µl DCFDA (10 µΜ), followed by incubation at 37 °C for 30 min. After incubation, solutions were aspirated, and cells were washed twice with PBS. The fluorescence signal of DCFDA was recorded with excitation at 485 nm and emission at 530 nm at 30 °C for 30 min. To evaluate changes in the mitochondrial membrane potential of SH-SY5Y cells, aliquots of incubated samples (20 µM) were added to cells, followed by incubation for 24 h. Then, the medium was replaced with 100 µL rhodamine 123 (1 µΜ), followed by incubation at 37 °C for 30 min. After incubation, solutions were aspirated, and cells were washed twice with PBS. The fluorescence signal of rhodamine 123 was recorded with excitation at 503 nm and emission at 527 nm at 30 °C for 30 min^67^. All fluorescence measurements were done in triplicate using a Synergy Hybrid Multi-Mode microplate reader (BioTek Instruments, Winooski, VT 05,404-0998, USA). At the end of incubation, 10 µL of incubated samples were added on a clean glass slide and then air-dried. The images were captured on a fluorescence microscope (Zeiss, Germany) at 20 × magnification.

### Hemolysis assay

Erythrocytes were separated by centrifugation of fresh blood samples at 1000 g for 10 min. The resulting pellets were collected and washed three times with PBS. To assess the protective effects of GTPs/GTPs-capped AgNPs against permeabilization induced by amyloid aggregates, erythrocytes were incubated alone or with various concentrations of incubated samples (20 µΜ) for 3 h at 37 °C with gentle stirring. Then, samples were centrifuged at 1000 g for 10 min, and the absorbance of supernatants at 540 nm was measured. The percentage of hemolysis was calculated as a fraction of the maximum effect (1% (v/v) Triton X-100) using the following formula:

[(measured signal-blank signal)/(maximum signal-blank signal)] × 100.

Erythrocytes treated with PBS were used as control. All assays were carried out in triplicate.

### Statistical analysis

All assays were performed two or three times with triplicate repeats. The data are presented as a percentage relative to values obtained from untreated control cells, and each value represents the mean ± SD (n = 3). Statistical significance was determined using an unpaired Student’s t- test, where *p < 0.01 indicates a significant difference compared to the control group, and #*p* < 0.01 indicates a significant difference compared to the group exposed solely to amyloid fibrils.

### Ethics statement

We confirm that all methods were performed in accordance with the relevant guidelines and regulations.

### Supplementary Information

Supplementary Information.

## Data Availability

The data that support the findings of this study are available from the corresponding author upon reasonable request.
