# Supplementary material for: Efficient inhibition of amyloid fibrillation and cytotoxicity of α-synuclein and human insulin using biosynthesized silver nanoparticles decorated by green tea polyphenols
Source: Sci Rep. 2024 Feb 16;14:3907. doi: 10.1038/s41598-024-54464-4 (PMC10873377; doi:10.1038/s41598-024-54464-4)
Supplement: Supplementary file 1 — Supplementary Information. [file 41598_2024_54464_MOESM1_ESM.docx]

**Supporting information**

**Efficient Inhibition of Amyloid Fibrillation and Cytotoxicity of α-Synuclein and Human Insulin Using Biosynthesized Silver Nanoparticles Decorated by Green Tea Polyphenols**

Behnaz Mirzaei-Behbahani^1^, Ali Akbar Meratan^1*^, Beitollah Moosakhani^1^, Mahya Mohammad-Zaheri^2^, Zahra Mousavi-Jarrahi^2^, Nasser Nikfarjam^3^, Mohammad Bagher Shahsavani^4^, Ali Akbar Saboury^2*^.

^1^ Department of Biological Sciences, Institute for Advanced Studies in Basic Sciences (IASBS), Zanjan 45137-66731, Iran.

^2^ Institute of Biochemistry and Biophysics, University of Tehran, Tehran 1417614335, Iran.

^3^ Department of Chemistry, Institute for Advanced Studies in Basic Sciences (IASBS), Zanjan 45137-66731, Iran.

^4^ Protein Chemistry Laboratory (PCL), Department of Biology, College of Sciences, Shiraz University, Shiraz 7196484334, Iran.

**
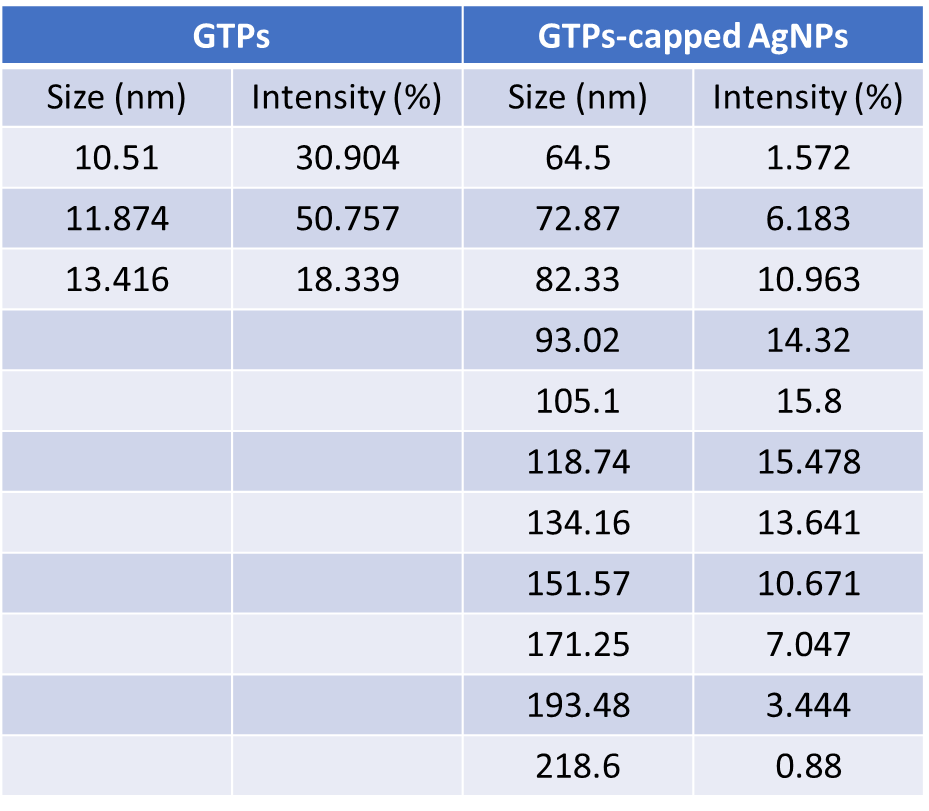
**

**Table S1.** Size and intensity pattern of GTPs and GTPs-capped AgNPs measured by DLS analysis.


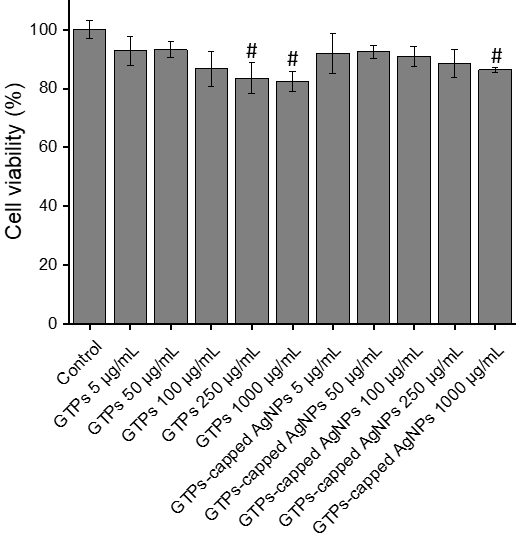


**Figure S1.** Dose-dependent toxicity of the GTPs and GTPs-capped AgNPs evaluated by MTT-based cell viability assay. ^#^p < 0.01, significantly different from control cells.


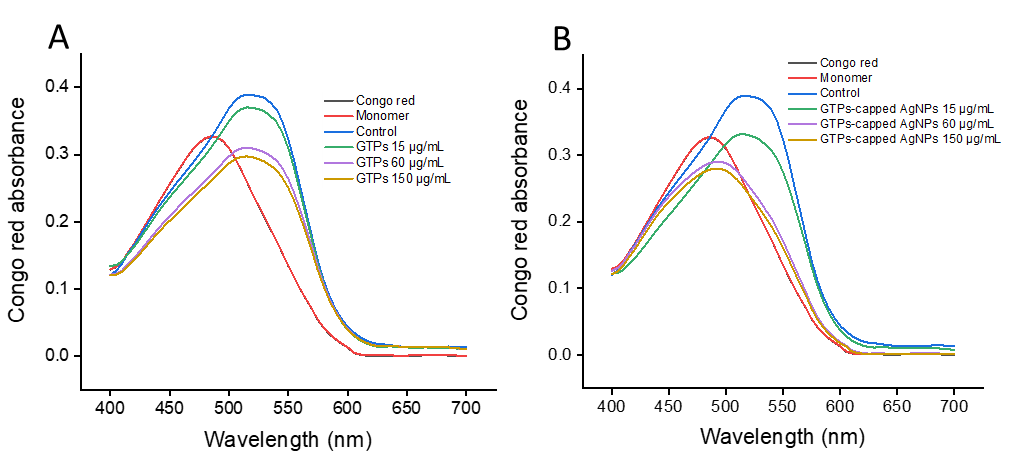


**Figure S2.** Congo red binding absorption spectra of human insulin in the absence and presence of various concentrations of (A) GTPs or (B) GTPs-capped AgNPs.


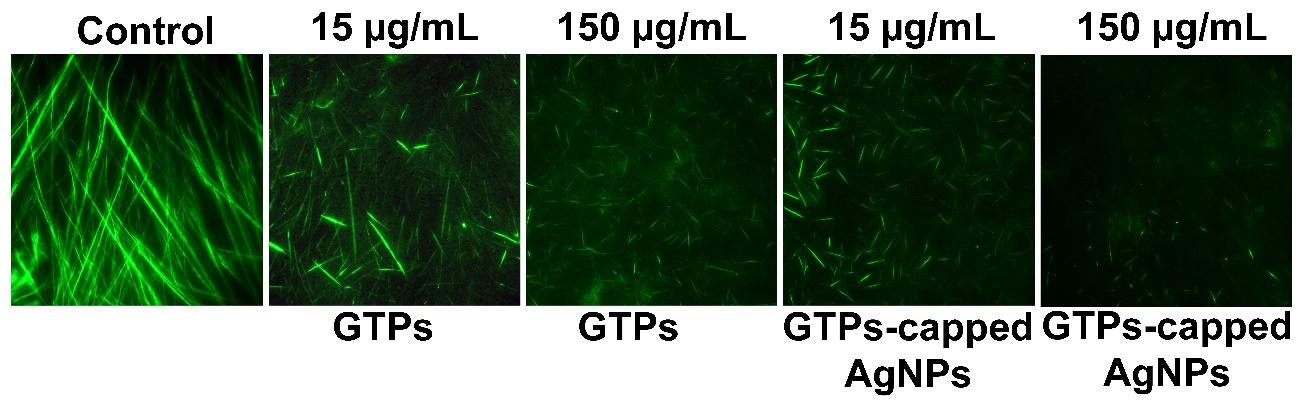


**Figure S3.** ThT fluorescence microscopy images of human insulin incubated without or with various concentrations of GTPs or GTPs-capped AgNPs under amyloidogenic conditions.

**
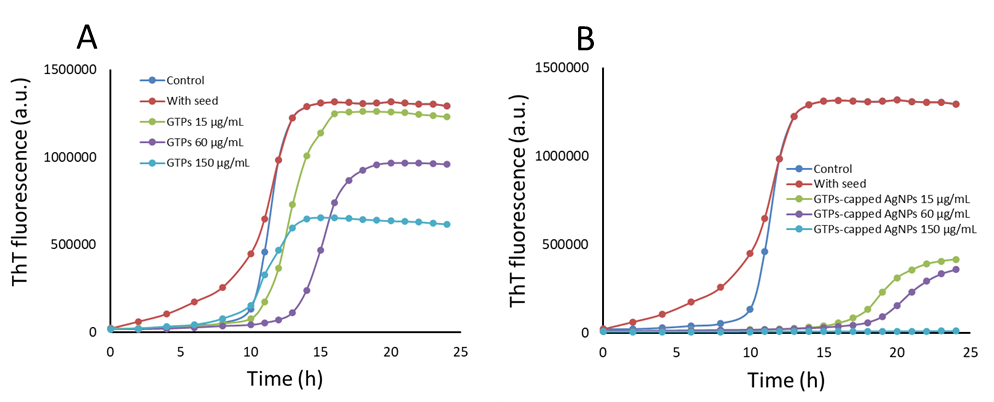
**

**Figure S4.** Effect of increasing concentrations of (A) GTPs and (B) GTPs-capped AgNPs on the secondary nucleation of human insulin fibrillization. Data are expressed as mean ± SD with n=3.


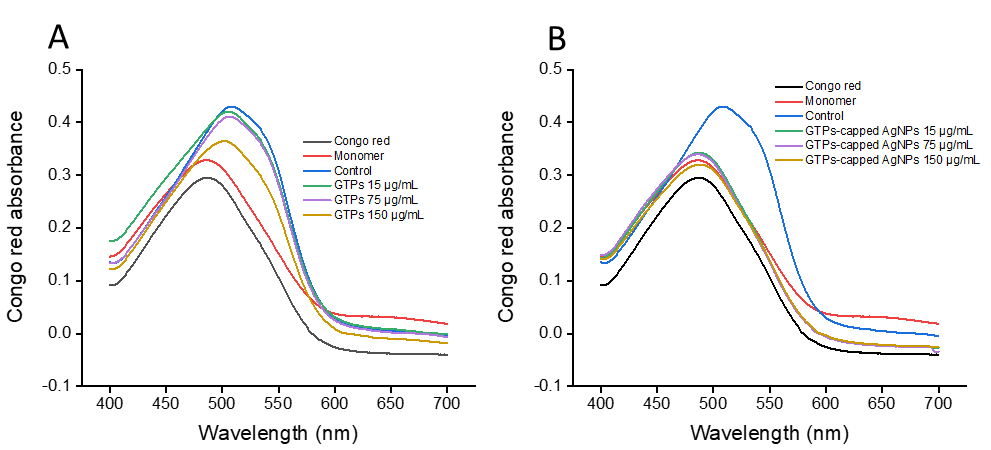


**Figure S5.** Congo red binding absorption spectra of α-syn in the absence and presence of various concentrations of (A) GTPs or (B) GTPs-capped AgNPs.

**
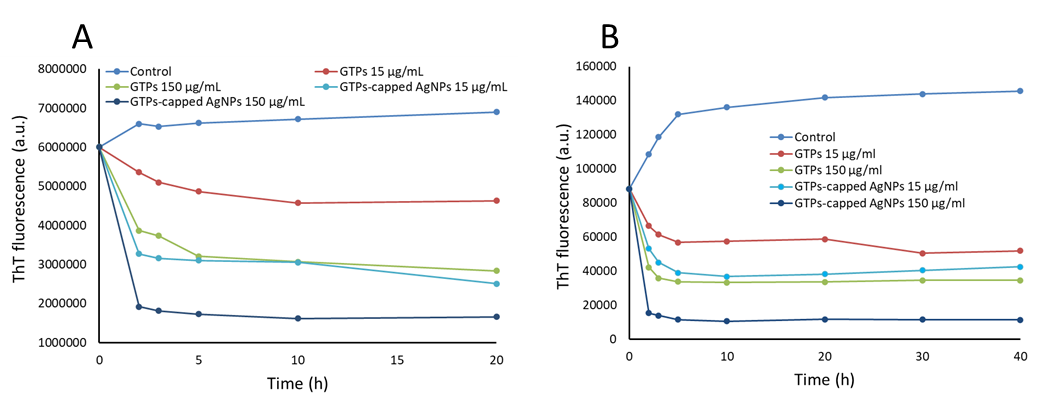
**

**Figure S6.** Remodeling of preformed fibrils of (A) bovine insulin and (B) α-syn by GTPs or GTPs-capped AgNPs demonstrated by decreasing fluorescence intensity of ThT at 485 nm. Preformed fibrils were incubated at 37 °C either alone or with various concentrations of GTPs or GTPs-capped AgNPs for 64 h.
